# A cohort profile of the Graham Roberts study cohort

**DOI:** 10.3389/fonc.2023.1334183

**Published:** 2024-01-09

**Authors:** Beth Russell, Poppy Leech, Harriet Wylie, Charlotte Louise Moss, Anna Haire, Deborah Enting, Suzanne Amery, Kathryn Chatterton, Muhammad Shamim Khan, Ramesh Thurairaja, Rajesh Nair, Sachin Malde, Kate Smith, Cheryl Gillett, Debra Josephs, Elias Pintus, Sarah Rudman, Simon Hughes, Clare Relton, Mieke Van Hemelrijck

**Affiliations:** ^1^ Translational Oncology and Urology Research, School of Cancer and Pharmaceutical Sciences, King’s College London, London, United Kingdom; ^2^ Urology, Guy’s and St Thomas’ NHS Foundation Trust, London, United Kingdom; ^3^ King’s Health Partners Cancer Biobank, King’s College London, London, United Kingdom; ^4^ Wolfson Institute of Population Health, Queen Mary University of London, London Sheffield, United Kingdom

**Keywords:** trials within cohorts, cohort profile, non-muscle invasive bladder cancer (NMIBC), muscle invasive bladder cancer (MIBC), questionnaires

## Abstract

**Purpose:**

The Graham Roberts Study was initiated in 2018 and is the first Trials Within Cohorts (TwiCs) study for bladder cancer. Its purpose is to provide an infrastructure for answering a breadth of research questions, including clinical, mechanistic, and supportive care centred questions for bladder cancer patients.

**Participants:**

All consented patients are those aged 18 or older, able to provide signed informedconsent and have a diagnosis of new or recurrent bladder cancer. All patients are required to have completed a series of baseline questionnaires. The questionnaires are then sent out every 12 months and include information on demographics and medical history as well as questionnaires to collect information on quality of life, fatigue, depression, overall health, physical activity, and dietary habits. Clinical information such as tumor stage, grade and treatment has also been extracted for each patient.

**Findings to date:**

To date, a total of 125 bladder cancer patients have been consented onto the study with 106 filling in the baseline questionnaire. The cohort is made up of 75% newly diagnosed bladder cancer patients and 66% non-muscle invasive bladder cancer cases. At present, there is 1-year follow-up information for 70 patients, 2-year follow-up for 57 patients, 3-year follow-up for 47 patients and 4-year follow-up for 19 patients.

**Future plans:**

We plan to continue recruiting further patients into the cohort study. Using the data collected within the study, we hope to carry out independent research studies with a focus on quality of life. We are also committed to utilizing the Roberts Study Cohort to set up and commence an intervention. The future studies and trials carried out using the Roberts Cohort have the potential to identify and develop interventions that could improve the prevention, diagnosis, and treatment of bladder cancer.

## Introduction

The Graham Roberts Study (Roberts Study), initiated in 2018, presents as the first Trials Within Cohorts (TwiCs) study for bladder cancer, designed to provide an infrastructure for answering a breadth of research questions, including clinical, mechanistic, and supportive care centred questions for bladder cancer patients (1). A detailed description of the study design has been published previously (1).

The study uses the TwiCs study design, introduced by Relton et al. (2), to obtain a cohort of patients with a new or recurrent diagnosis of bladder cancer. Patients are recruited at Guys and St Thomas’ (GSTT) National Health Service (NHS) Foundation Trust in London. Eligible patients have a basic understanding of the English language, are over the age of 18 and may have been referred to GSTT from secondary and tertiary hospitals across the UK.

All eligible patients who have already undergone diagnostic investigations and informed about a (highly likely) bladder cancer receive detailed written information about the Roberts Study while attending the Urology Centre for their initial appointment. The study uses a staged and tailored approach to informed consent. Prior to their first appointment with the consultant (urology or oncology) consent is sought to take part in the cohort and longitudinal study, to participate in the intervention arm of any future RCT, to be randomly selected to the control arm of any future RCT without further notification, to collection and storage of biological samples, including blood, urine and tissue, and linkage to routinely collected clinical data as recorded in electronic patient records.

The Graham Roberts Study collects health-related quality of life (HRQoL) patient-reported outcomes every twelve months using questionnaires that address: an assessment of bladder cancer illness, fatigue, depression, generic health status, physical activity, and diet quality.

The asymmetric informed consent nature of this study means that randomization can be proceeded by asymmetric treatment for two arms, where participants within the intervention arm will provide informed consent, and those in the control arm use the consent broadly disclosed before randomization. As such, the participants have a detailed understanding of prospective studies within the cohort before participation.

The purpose of this cohort profile is to describe the baseline demographic and medical history information for the patient cohort which have been recruited into the Roberts Study thus far including further information on their clinical and treatment characteristics.

## Cohort description

The first patient was consented onto the Graham Roberts Study on 7th March 2018. To date (October 2023), a total of 125 bladder cancer patients have been consented onto the study with all participants presenting a recent diagnosis of bladder cancer, whether this be a new diagnosis or a diagnosis of a recurrence. Nineteen patients did not complete the baseline questionnaire (15%). As completion of the baseline questionnaire was a pre-requisite for participation in this study, 106 consented patients were included in the study.

Information on the patients’ demographic information collected within the questionnaires includes age, sex, ethnicity, marital status, employment status, living arrangements and education level. Information regarding socioeconomic status was collected using the English indices of deprivation 2019 online tool (3). Postcodes were converted into indices of multiple deprivation (IMD) deciles. The IMD deciles were then converted to quintiles, with 1 being the most deprived and 5 being the least deprived.

Information on the medical history of the patients collected using the questionnaires includes their personal history with cancer and any family history with cancer (including both all cancers and bladder cancer specifically). Furthermore, information on bladder cancer related symptoms is collected such as history of nephritic colic, or kidney, renal or bladder stones, bladder or kidney infections, issues with urination and any history of an enlarged prostate (for the males only).

As well as the questionnaires on demographical factors and medical history, information was collected on the patients’ alcohol and smoking habits. Further to this, patient reported outcomes (PROs) have been collected utilizing the following validated questionnaires:

- *QoL*: Functional Assessment of Chronic Illness Therapy for Bladder Cancer (4).
- *Fatigue*: Functional Assessment of Chronic Illness Therapy-Fatigue (5).
- *Depression*: Patient Health Questionnaire-9 (6).
- *Health*: Standardized instrument for use as a measure of health outcome (EQ-5D-5L10) (7).
- *Physical activity*: Short Questionnaire to Assess Health-Enhancing Physical Activity (8).
- *Assessment of dietary habits*: Short Questionnaire to Assess Diet Quality (9).

The items were collected as 5-point Likert scales, objective responses, and free-text answers. A summary of the items collected is outlined in **Table 1**. Due to the large quantity of data points a summary of this information is out of the scope of this current cohort profile.

**Table 1:** Description of PROMs collected within the Graham Roberts Study.

Functional Domain	Validated Questionnaire	Item summary
***Quality of Life***	Functional Assessment of Chronic Illness Therapy for Bladder Cancer	• Physical wellbeing • Social/family wellbeing • Emotional wellbeing • Functional wellbeing
***Fatigue***	Functional Assessment of Chronic Illness Therapy Fatigue Subscale	• Coping with everyday activities • Fatigue and weakness
***Depression***	Patient Health Questionnaire 9	• Appetite changes • Trouble focussing • Negative thoughts
***Health***	A standardised instrument for use as a measure of health outcome (EQ-5D-5L)	• Mobility • Self-care • Usual activities • Pain/discomfort • Anxiety/depression
***Physical Activity***	Short Questionnaire to Assess Health Enhancing Physical Activity	• Walking • Bicycling • Other physical activities and steps
***Assessment of dietary habits***	Short Questionnaire to Assess Diet Quality	• Foods consumed • Milk consumption • Sugar intake

### Clinical and treatment information

No clinical information is collected directly from patients via the Roberts study questionnaires themselves. In order to collate this information for the cohort profile, the electronic health records for each patient are searched to extract the baseline clinical and treatment information for all study participants. Clinical staging information collected included tumor (T), nodal (N) and metastases (M) stages identified within imaging, the staging from the transurethral resection of the bladder tumor (TURBT) as well as the grade and CIS involvement. Treatment information extracted included the treatment paradigm such as radical, surveillance, neo-adjuvant and palliative, and the treatment modality such as cystectomy, systemic chemotherapy, laser ablation, immunotherapy, radiotherapy, intravesical therapy and neoadjuvant chemotherapy (NAC) with cystectomy.

### Follow-up information

**Figure 1:**
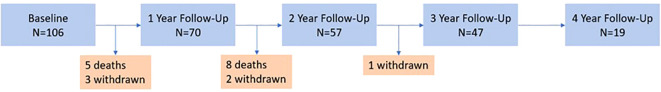
Survey population response rates.

The questionnaires are sent to all eligible consented patients every 12 months. At the point of consent, patients give their preference as to whether they would prefer the questionnaires to be sent via email or in the post. Given the study was initiated in 2018, at present, there is a maximum of four successive follow-ups. Throughout this study time frame, 13 (12%) patients have died, and 6 (6%) have withdrawn consent. As shown in **Figure 1**, we have 1-year follow-up information for 70 patients, 2-year follow-up for 57 patients, 3-year follow-up for 47 patients and 4-year follow-up for 19 patients.

### Baseline data of cohort

#### Cohort demographics

Overall 106 patients completed the baseline questionnaire, most of which (79%) were male (**Table 2**). In terms of ethnicity 88% of study participants were While/Caucasian, 6% Black/Afro-Caribbean, 1% Asian and 4% other ethnic backgrounds. The majority of patients were married (55%) and/or lived with a partner (66%). Only 9% of patients were in the first (most deprived) quintile, with the majority of patients either being in the second (29%) or third (26%) quintile. When asked about their employment status, 62% were retired and 32% were in some sort of full or part time work. Two thirds of the study participants reported to have been a regular smoker at some point in their life (66%).

**Table 2:** Demographic baseline characteristics of the Roberts study cohort.

	N	%
Sex		
Female	22	20.80
Male	84	79.20
Ethnicity		
White/Caucasian	93	87.70
Black/Afro-Caribbean	6	5.70
Asian	1	0.90
Other	4	3.80
Unknown	2	1.90
Marital status		
Married	58	54.70
Divorced/separated	12	11.30
Widowed	23	21.70
Never Married	11	10.40
Unknown	2	1.90
IMD (quintile)		
1	9	8.50
2	31	29.20
3	27	25.50
4	16	15.10
5	20	18.90
Unknown	3	2.80
Living arrangement		
Living alone	27	25.50
Living with partner	70	66.00
Living with other family	7	6.60
Other	1	0.90
Unknown	1	0.90
Employment status		
Full-time	22	20.80
Part-time	13	12.30
Retired	66	62.30
Disabled	1	0.90
Unemployed	3	2.80
Unknown	1	0.90
Education level		
Primary School	1	0.90
Secondary School	54	50.90
Higher Education (e.g. University)	41	38.70
Other	7	6.60
Unknown	3	2.80
Did you ever smoke regularly (at least one per day for six months or longer)?		
No	35	33.00
Yes	70	66.00
Unknown	1	0.90

#### Medical history

One quarter (24%) of study participants reported a history of cancer, 58% said they had a family history of cancer and 9% a family history of bladder cancer (**Table 3**). With regards to bladder cancer symptoms, 39% had a history of bladder infections with the highest proportion of these stating they had experienced the infection 1-2 times (39%). Most patients did not have a history of nephritic colic, or kidney, renal or bladder stones. The majority of patients did however say they experienced a difficulty in starting or stopping urinating or an increased frequency of urinating during the night (56%). Of the 84 men in the study, 29% reported to have had a history of an enlarged prostate.

**Table 3:** Medical History information of the Roberts Study Cohort.

	N	%
History of cancer		
No	76	71.70
Yes	25	23.60
Unknown	5	4.70
Family history of cancer		
No	42	39.60
Yes	61	57.50
Unknown	3	2.80
Family History of Bladder Cancer		
No	49	46.20
Yes	10	9.40
Unknown	47	44.30
History of bladder infection		
No	61	57.50
Yes	41	38.70
I don't know	3	2.80
Unknown	1	0.90
Bladder infection frequency (n=41)		
1-2 times	16	39.00
3-5 times	10	24.40
6-10 times	5	12.20
11 or more times	4	9.80
I don't know	4	9.80
Unknown	2	4.90
Kidney infection history		
No	93	87.70
Yes	8	7.50
I don't know	3	2.80
Unknown	2	1.90
Kidney infection frequency (n=8)		
1-2 times	4	50.00
11 or more times	3	37.50
Unknown	1	12.50
Before 1 year ago, did you ever have renal or nephritic colic, or kidney or renal stones?		
No	89	84.00
Yes	12	11.30
I prefer not to answer	1	0.90
I don't know	2	1.90
Unknown	2	1.90
History of urinary bladder stones?		
No	101	95.30
Yes	1	0.90
I don't know	1	0.90
Unknown	3	2.80
Before 1 year ago, did you ever have a growth removed from your urinary bladder?		
No	87	82.10
Yes	16	15.10
Unknown	3	2.80
Did you ever have any of the following symptoms when urinating: difficult starting, difficulty stopping or increased frequency during the night?		
No	39	36.80
Yes	59	55.70
I don't know	1	0.90
Unknown	7	6.60
History of enlarged prostate (n=84)		
No	57	67.90
Yes	24	28.60
I don't know	1	1.20
Unknown	2	2.40

#### Clinical characteristics of the cohort

When looking at the clinical characteristics of the study cohort, 75% of the patients recruited into the Roberts study were newly diagnosed with bladder cancer, whilst 25% were patients who had been diagnosed with recurrent bladder cancer (**Table 4**). Two thirds of patients had non-muscle invasive disease (66%) with the remaining patients diagnosed with muscle invasive (33%). From the imaging, only a very small proportion of patients were metastatic (4%) and/or had lymph node involvement (9%). Just under half of the patients had carcinoma *in situ* (CIS) (46%) and most patients were deemed to have high grade tumors (89%).

**Table 4:** Clinical Characteristics of the Roberts Study Cohort .

	N	%
Recurrence or Newly Diagnosed		
Newly Diagnosed	79	74.50
Recurrence	27	25.50
T Stage (Imaging)		
T1	3	2.80
T2	7	6.60
T3	5	4.70
T4	1	0.90
Ta	4	3.80
Tx	1	0.90
Unknown	85	80.20
N Stage (Imaging)		
N0	90	84.90
N1	7	6.60
N2	2	1.90
Unknown	7	6.60
M Stage (Imaging)		
M0	95	89.60
M1	4	3.80
Unknown	7	6.60
Stage (TURBT)		
Muscle Invasive	35	33.00
Non-muscle Invasive	71	66.90
T Stage (TURBT)		
Ta	36	34.00
T1	32	30.20
T2	27	25.50
T3	5	4.70
T4	2	1.90
Unknown	4	3.80
CIS		
No	49	46.20
Yes	41	38.70
Unknown	16	15.10
Grade		
High Grade	94	88.70
Low Grade	12	11.30

#### Bladder cancer treatments

**Figure 2:**
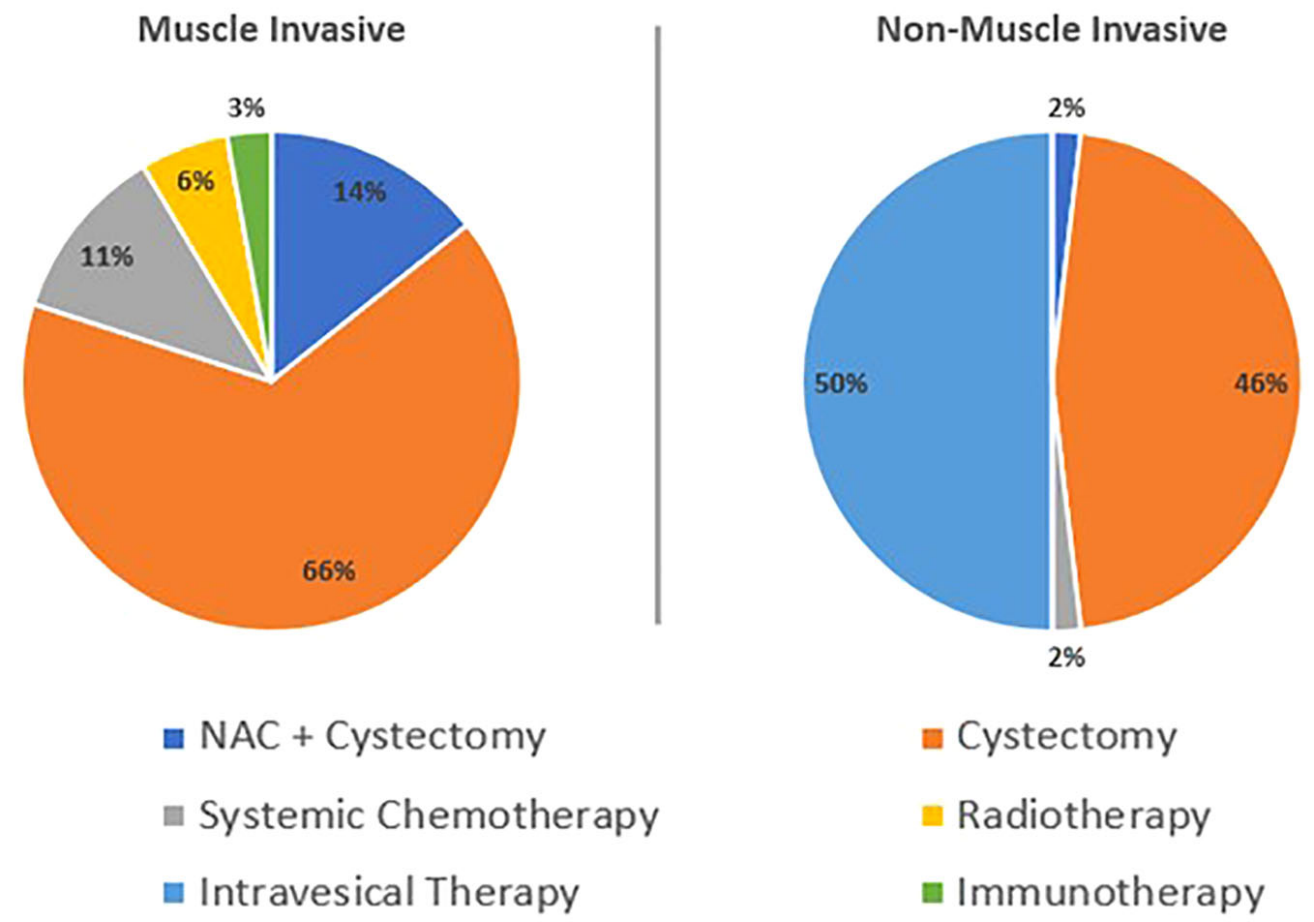
Treatment by Non-Muscle Invasive Bladder Cancer and Muscle Invasive Bladder Cancer.

When appraising treatment paradigm, most patients were undergoing radical or curative treatments (78%) (**Table 5**). Fifteen percent of patients were under surveillance with a small proportion undergoing palliative (3%) or neoadjuvant therapy (3%). The treatments by NMIBC and MIBC are depicted in **Figure 2**. Of the MIBC patients, the overwhelming majority had undergone a cystectomy (66%), with a further 14% having undergone neoadjuvant chemotherapy (NAC) and a cystectomy. A small proportion of patients had received systemic chemotherapy (11%), or radiotherapy (6%) and one patient had received immunotherapy (3%). In the NMIBC cohort, of those undergoing active treatment, half of the patients had undergone intravesical therapy (the full breakdown of which can be found in **Table 5**) and almost half had undergone a cystectomy (46%). The remaining patients had either undergone NAC and a cystectomy (2%) or systemic chemotherapy (2%).

**Table 5:** Treatment information for the Roberts Study Cohort.

	N	%
Treatment Paradigm		
Neoadjuvant	3	2.80
Palliative	3	2.80
Radical/Curative	83	78.30
Surveillance	16	15.10
Unknown	1	0.90
**NAC and Cystectomy**	6	5.70
**Cystectomy**	48	46.20
**Systemic Chemotherapy**	5	4.70
**Radiotherapy**	2	1.90
Intravesical Therapy		
BCG	10	9.40
Di Stasi	15	14.20
Mitomycin	1	0.90
Other	1	0.90
Unknown	79	74.50
**Immunotherapy**	1	0.90

### Plans for future studies

The Graham Roberts study provides a unique opportunity to conduct multiple pragmatic trials within the cohort. One advantage of the trials within cohorts design is that that patients randomly assigned to usual care do not need to be recontacted as they have already given broad consent prior to randomization. The Graham Roberts study collects a wide range of PROMs on quality of life and lifestyle factors including diet and physical activity. When paired with the clinical information, this gives the opportunity to look at potential associations between clinical factors and treatments with quality-of-life outcomes. This provides the opportunity to address a wide range of research questions from one cohort, a strength that will only continue to grow over time as more patients recruited and longer-term follow-up data is accrued.

Despite running since 2018, we are yet to initiate a trial with a TwiCS design within the Roberts Study. This is mainly due to the COVID-19 pandemic, which, like many studies, put a halt to both recruitment and progression of the study. Furthermore, the recruitment target is somewhat below the original target set out in our first paper (1), owing to the interruption of services and an inability to recruit patients during the pandemic. From previous experiences, we are also aware that introducing interventions can often take longer than first planned (10). However, we are committed to the introduction and initiation of a TwiCS trial in the near future. Our focus for this is likely to be in the mental wellbeing space given the growing interest and emphasis on this important research area (11).

### Strengths and limitations

We acknowledge that the number of study participants decreases with each follow-up for reasons other than death or withdrawals from the study. One is that as patients are still being recruited into the study, not all patients have reached each follow-up yet. Another reason is that patients don’t always return their questionnaires straight away or sometimes at all. To address this decrease in adherence, the study team will start sending reminders to patients to fill in their questionnaires. During this process, we will also try and gain an understanding as to why patients are no longer returning their questionnaires and will work on solutions to resolve this. The cohort of patients so far included within the Roberts study are representative of the bladder cancer patients seen at our institution in South-East London. Furthermore, the TwiCS trial design presents several advantages over the traditional randomized controlled trial (RCT) method. Firstly, it addresses the difficulties in recruiting patients onto trials, by capitalizing on the nature of observational cohort studies being easier and less selective (12). Additionally, it means that prospective trials within the cohort do not require further recruitment efforts. In fact, a recent study revealed that recruitment for RCTs within these established cohorts is more efficient when compared with traditional recruitment without cohorts (13). A further advantage of the TwiCs study design is how obtaining informed consent can be tailored to the relevant needs of each participant. As such the TwiCS design is patient-centred, offering a process for gaining informed consent that mirrors the real-world routine in healthcare, where patients are not told about treatments that they are not offered.

## Conclusions

The Roberts Study is the first study within bladder cancer using a ‘trials within cohorts’ study design. The data collected within the study is broad and rich and can be used independently to conduct research studies, including studies exploring the quality of life. We hope that this study will help identify and develop interventions to improve the prevention, diagnosis, and treatment of bladder cancer in the future.

## Data availability statement

The datasets presented in this article are not readily available due to patient anonymity. Requests to access the datasets should be directed to the corresponding author.

## Ethics statement

The studies involving humans were approved by London–Fulham Research Ethics Committee as part of gaining Health Research Authority approval (17/LO/1975). The studies were conducted in accordance with the local legislation and institutional requirements. Written informed consent for participation was not required from the participants or the participants’ legal guardians/next of kin in accordance with the national legislation and institutional requirements.

## Author contributions

BR: Conceptualization, Data curation, Formal analysis, Methodology, Writing – original draft, Writing – review & editing. PL: Data curation, Writing – review & editing. HW: Data curation, Project administration, Validation, Writing – review & editing. CM: Project administration, Validation, Writing – review & editing. AH: Data curation, Project administration, Validation, Writing – review & editing. DE: Data curation, Writing – review & editing. SA: Writing – review & editing. KC: Writing – review & editing. MK: Writing – review & editing. RT: Writing – review & editing. RN: Writing – review & editing. SM: Writing – review & editing. KS: Writing – review & editing. CG: Writing – review & editing. DJ: Writing – review & editing. EP: Writing – review & editing. SR: Writing – review & editing. SH: Writing – review & editing. CR: Writing – review & editing. MV: Supervision, Writing – review & editing.
